# Hexathioalkyl sumanenes: an electron-donating buckybowl as a building block for supramolecular materials†
†Electronic supplementary information (ESI) available: Experimental and crystallographic details, analytical data and theoretical calculations. CCDC 1572348 and 1572349. For ESI and crystallographic data in CIF or other electronic format see DOI: 10.1039/c7sc03860g


**DOI:** 10.1039/c7sc03860g

**Published:** 2017-10-18

**Authors:** Yoshiaki Shoji, Takashi Kajitani, Fumitaka Ishiwari, Qiang Ding, Hiroyasu Sato, Hayato Anetai, Tomoyuki Akutagawa, Hidehiro Sakurai, Takanori Fukushima

**Affiliations:** a Laboratory for Chemistry and Life Science , Institute of Innovative Research , Tokyo Institute of Technology , 4259 Nagatsuta, Midori-ku , Yokohama 226-8503 , Japan . Email: fukushima@res.titech.ac.jp; b RIKEN SPring-8 Center , 1-1-1 Kouto, Sayo , Hyogo 679-5148 , Japan; c Rigaku Corporation , Matsubara-cho 3-9-12, Akishima , Tokyo 196-8666 , Japan; d Graduate School of Engineering , Tohoku University , Sendai 980-8579 , Japan; e Institute of Multidisciplinary Research for Advanced Materials (IMRAM) , Tohoku University , 2-1-1 Katahira, Aoba-ku , Sendai , 980-8577 , Japan; f Division of Applied Chemistry , Graduate School of Engineering , Osaka University , 2-1 Yamada-oka, Suita , Osaka 565-0871 , Japan

## Abstract

Unlike planar aromatic compounds, bowl-shaped sumanene, which has concave and convex faces with different electrostatic potentials, tends to form a one-dimensional columnar assembly without causing slip-stacking in the crystal. Here we report the first successful synthesis of liquid-crystalline (LC) sumanenes, which was brought about by the incorporation of six thioalkyl groups (R = SC_6_H_13_ or SC_12_H_25_) into the aromatic part of sumanene. In contrast to the case of the mesophase formation of corannulene, which requires the presence of many dendritic side chains, sumanene derivatives with simple alkyl chains can exhibit a remarkably high-order columnar LC mesophase over a wide temperature range. While non-substituted sumanene inherently behaves as an electron acceptor, hexathioalkyl versions, such as hexathiomethyl sumanene, show electron-donating properties, resulting in complexation with C_60_. Considering its unique shape, electronic properties, and self-assembly behavior, the electron-donating sumanene may represent a new building block for constructing supramolecular materials, both by itself and in combination with fullerene derivatives.

## Abstract 2

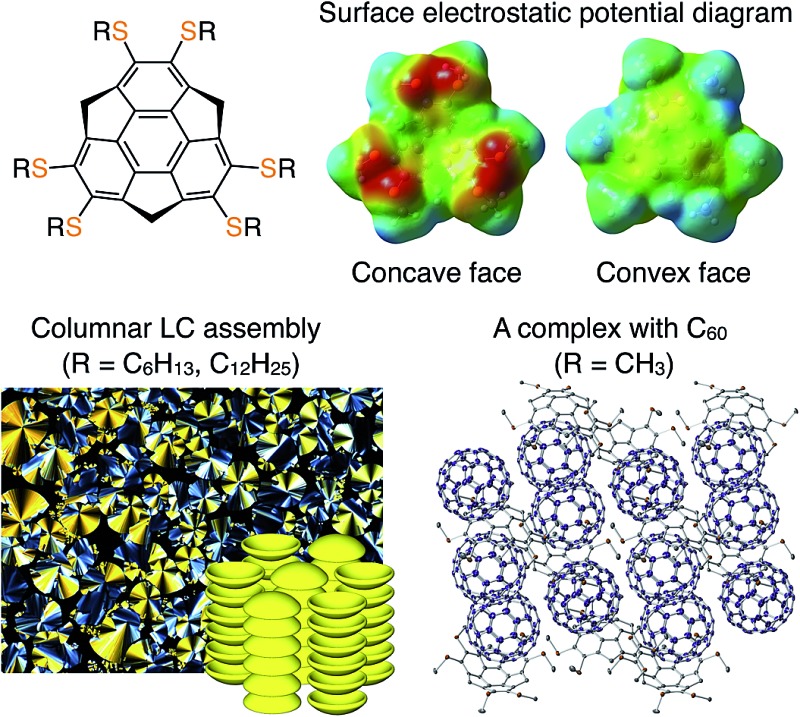
The synthesis and assembly behavior of hexathioalkyl sumanenes, having a different feature of surface electrostatic potential from non-substituted sumanene, are described.

## Introduction

**Fig. 1:**
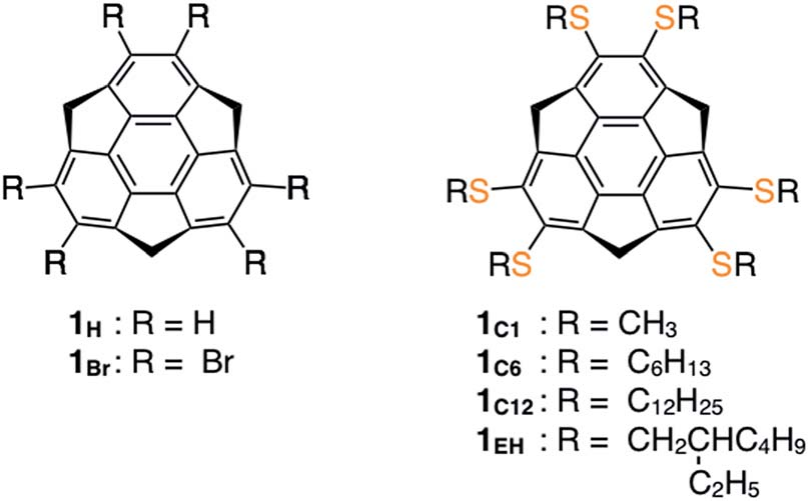
Molecular structures of sumanenes derivatives.

Buckybowls, which exhibit a concave–convex geometry, are promising molecular building blocks for the development of functional organic materials and devices.^1^–^4^ Sumanene C_21_H_12_ (**1_H_**, Fig. 1) is a representative buckybowl and is characterized by a *C*_3v_-symmetric subunit of C_60_, *i.e.*, a curved methylene-bridged triphenylene framework.^1^ In the crystal, **1_H_** assembles through a concave–convex interaction^5^ to form a one-dimensional (1D) bowl-stack column without slip-stacking geometry.1*b* In contrast, this structural pattern is not observed in the crystal of corannulene C_20_H_10_, which is another type of buckybowl.^2^ According to previous reports, the construction of bowl-stacked 1D assemblies of corannulene should require proper structural extensions of its π-conjugated framework2d,e,i,j or elaborate side chains with strong hydrogen-bonding capability.2f,g Based on the difference between these two buckybowls with respect to self-assembly behavior, sumanene, with a strong preference for bowl stacking, is advantageous for the design of a highly ordered 1D columnar architecture.

Another interesting feature of sumanene is that it exhibits bowl-to-bowl inversion, which is associated with dipole inversion.^1^ If a collective bowl-to-bowl inversion could be induced for columnarly assembled sumanene through the application of electric fields, dielectric responsive properties such as ferroelectricity could be realized.^6^ For this purpose, liquid-crystalline (LC) assemblies are expected to have great advantage over crystalline assemblies. However, LC sumanene has not yet been developed.

Here we report the first sumanene derivatives that can form LC mesophases with a highly ordered hexagonally arranged columnar structure over a wide temperature range. The LC sumanenes carry six simple thioalkyl side chains at the peripheral aromatic positions (**1_C6_** and **1_C12_**, Fig. 1), which is in striking contrast to LC corannulenes.2f,g The single-crystal X-ray analysis of **1_C1_** with six thiomethyl groups (Fig. 1) showed a 1D bowl-stacking structure. Theoretical calculations suggest that hexathioalkyl sumanenes have an inversed and enhanced concave–convex polarization of the sumanene core, compared to that of non-substituted **1_H_**. We also demonstrate that **1_C1_** behaves as an electron donor due to the six thioalkyl substituents and forms a complex with C_60_, which has never been achieved with non-substituted sumanene (**1_H_**) or previously reported sumanene derivatives.

## Results and discussion

### Synthesis of hexathioalkyl sumanenes

Mainly due to the lack of a synthetic method for the full peripheral functionalization of sumanene,^1^ a sumanene derivative that can form an LC assembly has not yet been reported. Recently, we reported the successful synthesis of 2,3,5,6,8,9-hexabromosumanene (**1_Br_**, Fig. 1), which can readily be converted into hexa-arylated sumanene derivatives through Pd-catalyzed cross-coupling such as Suzuki–Miyaura reaction.1*i* We found that compound **1_Br_** also allows aromatic nucleophilic substitution with thioalkoxides. Typically, **1_Br_** was reacted with 18 equivalents of sodium thiomethoxide (NaSCH_3_) in 1,3-dimethyl-2-imidazolidinone (DMI) at 100 °C under argon to give **1_C1_** in 35% yield (ESI†). Similarly, **1_C6_**, **1_C12_** and **1_EH_** (Fig. 1) were obtained in 39–43% yields using the corresponding sodium thioalkoxide in place of NaSCH_3_ (ESI†). All of these hexathioalkyl sumanenes were unambiguously characterized by ^1^H and ^13^C NMR spectroscopy, IR spectroscopy, and high-resolution APCI-TOF mass spectrometry (ESI†). For instance, the ^1^H NMR spectrum of **1_C1_** in toluene-*d*_8_ at 25 °C showed two doublet signals at *δ* = 4.50 and 3.54 ppm arising from the benzylic protons at the *exo*- and *endo*-positions, respectively (Fig. S1, ESI†). Note that these signals were not coalesced, even at elevated temperatures (*e.g.*, 100 °C, Fig. S1, ESI†). Thus, it is likely that the rate of bowl-to-bowl inversion of **1_C1_** in solution is sufficiently slow relative to the timescale of ^1^H NMR spectroscopy.

### X-ray crystal structure of hexathiomethyl sumanene (**1_C1_**)

**Fig. 2:**
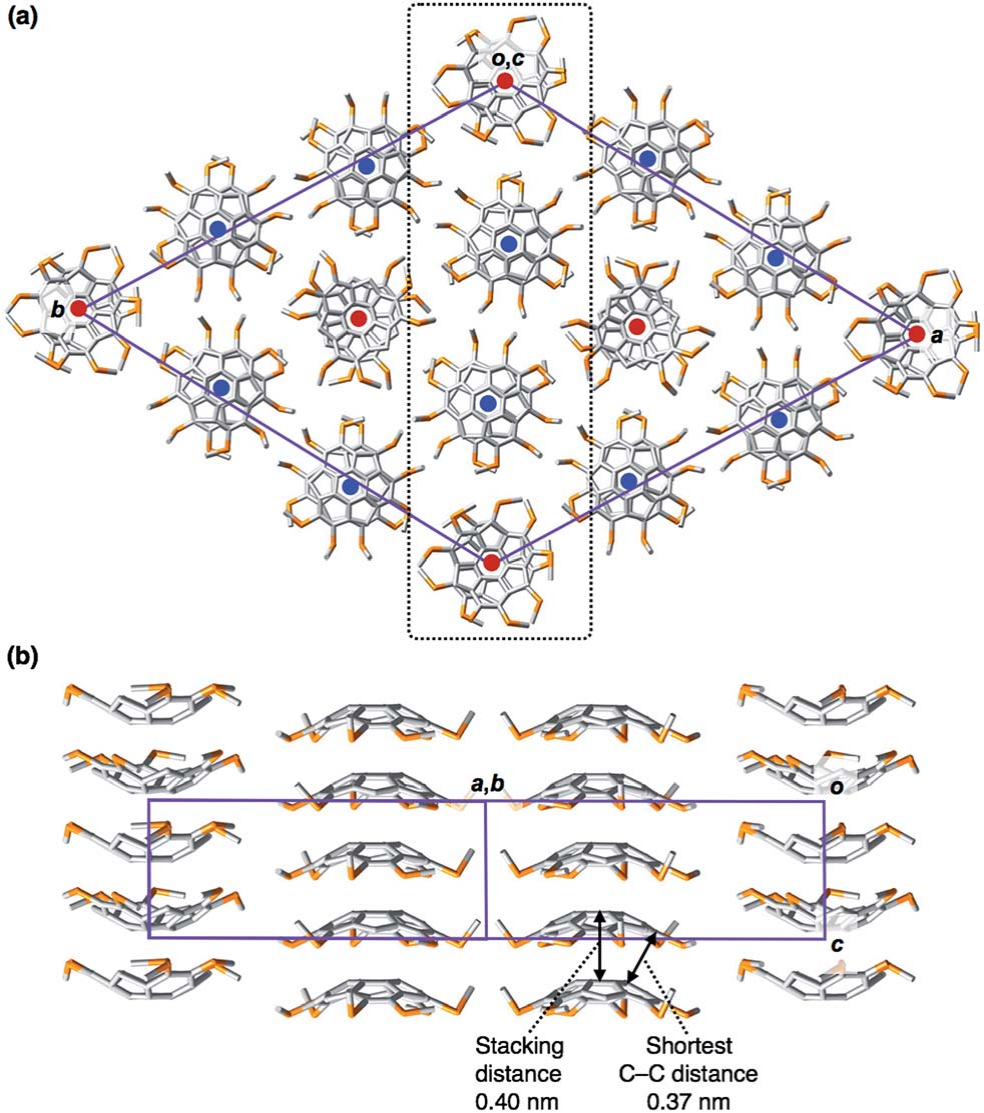
(a) Crystal packing diagram of **1_C1_** viewed along the *c* axis. In the 1D columns indicated with blue circles, **1_C1_** molecules pile up in such a way that the convex face is oriented to the front side, while the 1D columns indicated with red circles consist of **1_C1_** with an opposite concave orientation. (b) Packing diagram of 1D columns of **1_C1_** viewed along the (1–10) plane, which corresponds to the part surrounded by a broken line in (a).

We successfully obtained needle-shaped pale-yellow single crystals of **1_C1_**, suitable for X-ray diffraction analysis, by the slow diffusion of a hexane vapor into a dichloromethane solution of **1_C1_** (ESI†). Single-crystal X-ray analysis revealed detailed molecular and assembled structures of **1_C1_**. The crystal of **1_C1_** belongs to the *P*3 space group, and the asymmetric unit in the unit cell contains four entire **1_C1_** molecules and six fragments of one-third of **1_C1_** (*Z* = 18). Overall, each crystallographically independent molecule is very similar in terms of bond lengths and angles. The mean bowl-depth of **1_C1_** (1.04 Å, Fig. 2) at the peripheral aromatic carbons is slightly shallower than those observed for the crystal structures of **1_H_** (1.11 Å)1*a* and **1_Br_** (1.08 Å).1*i* In the crystal of **1_C1_**, 1D columns are formed in a bowl-stack manner with a quasi-staggered stacking geometry, where the mean stacking distance (4.00 Å) is comparable to that observed for **1_Br_** (3.94 Å),1*i* while it is longer than that observed for **1_H_** (3.86 Å).1*a* Considering the observed stacking geometry of **1_C1_**, along with the previously reported simulation for **1_H_**,^7^ an intermolecular electrostatic force is likely responsible for the formation of the 1D column. Meanwhile, the 1D columns assemble laterally to form a 2D quasi-hexagonal structure with an inter-columnar distance of approximately 12 Å (Fig. 2). When viewed along the *c*-axis, six columns with a convex upward geometry surround a column with a convex downward geometry, so that the dipole moments generated in the column can be partially cancelled (Fig. 2a). The 2D quasi-hexagonal structure developed in the crystal of **1_C1_** is reminiscent of the structure of a hexagonal columnar (Col_h_) mesophase that is typically observed for discotic LC assemblies.^8^

### DFT calculations of the molecular and electronic structures of hexathiomethyl sumanene (**1_C1_**)

**Fig. 3:**
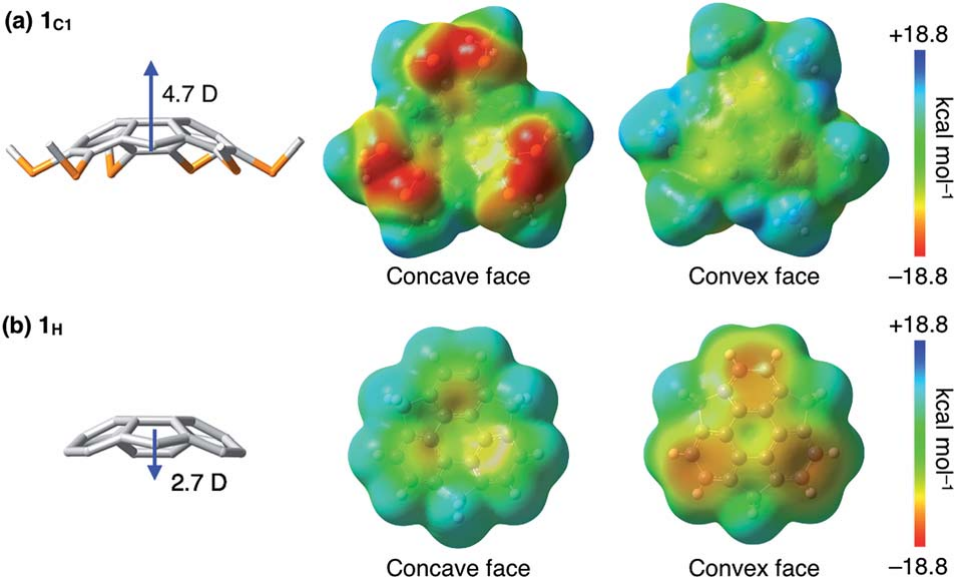
Calculated electric dipole moments and electrostatic potential surfaces of (a) **1_C1_** and (b) non-substituted **1_H_** at the w97BD/6-311G++(d,p) level of theory. The blue arrows indicate the directions of the dipole moments.

To gain insight into how the thioalkyl groups affect the electronic structure of sumanene, we performed density functional theory (DFT) calculations on the isolated molecule of **1_C1_** in vacuum at the w97BD/6-311G++(d,p) level (Fig. 3 and Table S1, ESI†). The molecular structure of **1_C1_** obtained by the single-crystal X-ray analysis was used as the initial geometry for geometry optimization. The calculated bond lengths and angles in the optimized geometries were in good agreement with those observed for the crystal structure of **1_C1_** (Fig. 2). Because of the electron-rich sulfur atoms, the calculated dipole moment of **1_C1_** along its *C*_3_-symmetric axis [4.7 Debye (D)] was much greater than that calculated for non-substituted sumanene **1_H_** (2.7 D) at the same level (Table S2, ESI†), and the directions of the dipole moments of **1_C1_** and **1_H_** were opposite to one another (Fig. 3). The surface electrostatic potential (ESP) diagram of **1_C1_** showed negative and positive ESP values for the concave and convex faces, respectively (Fig. 3). We suppose that intermolecular electrostatic attractive interactions between positive convex and negative concave faces of **1_C1_** could compensate for the unfavorable parallel dipole alignment, leading to the formation of the columnar structure in the crystal (Fig. 2). Note that, since the ESP diagram of **1_H_** illustrates negative convex and relatively positive concave faces (Fig. 3),1*l* the six thioalkyl groups in **1_C1_** invert and enhance the concave–convex polarization of the sumanene skeleton.

### Characterization of liquid-crystalline (LC) mesophases of sumanene derivatives

**Fig. 4:**
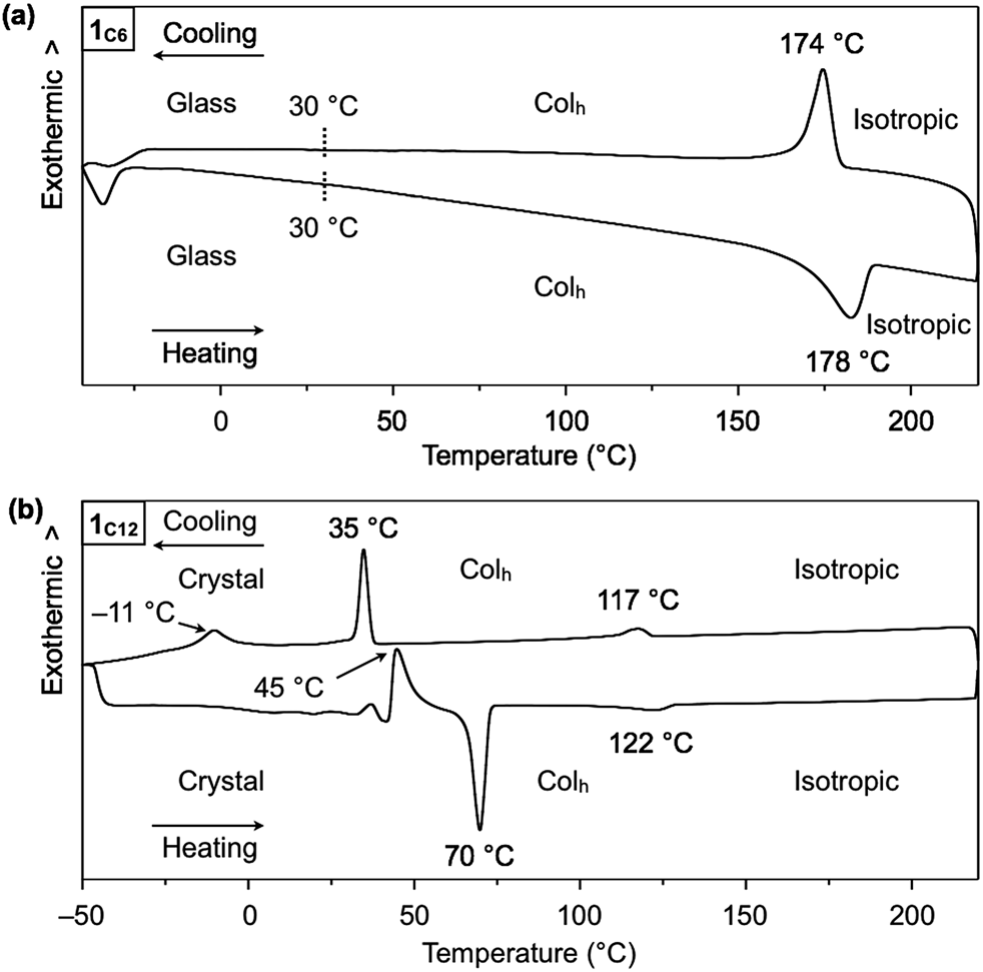
DSC traces of (a) **1_C6_** and (b) **1_C12_** on second heating and cooling (scan rate = 10 °C min^–1^).

**Fig. 5:**
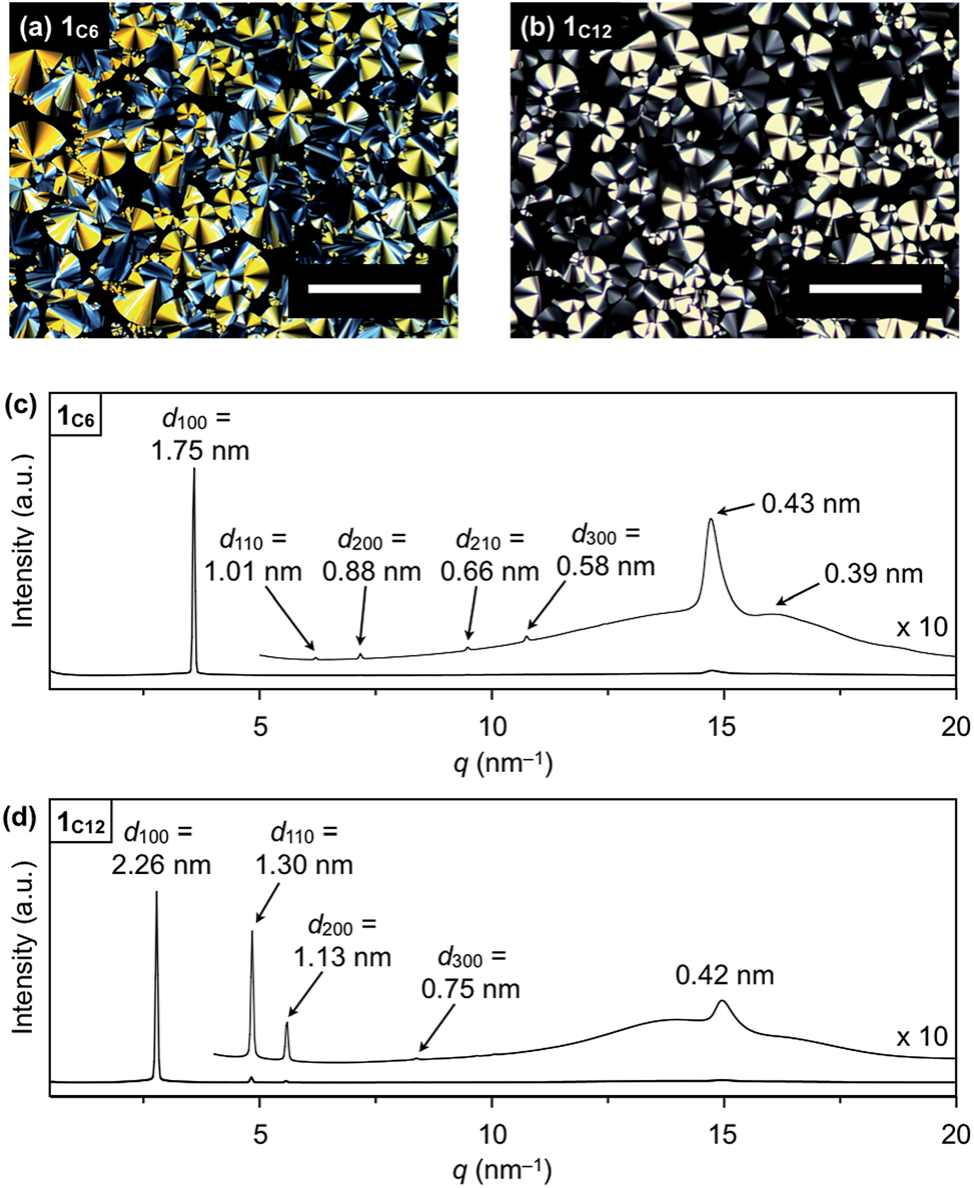
POM images of (a) **1_C6_** at 140 °C on cooling and (b) **1_C12_** at 80 °C on cooling (scale bar = 200 μm). XRD patterns of (c) **1_C6_** and (b) **1_C12_** in a glass capillary at 100 °C on heating.

Hexathioalkyl sumanenes with long alkyl side chains (**1_C6_** and **1_C12_**, Fig. 1) exhibit bowl-stacking to form a highly ordered columnar LC assembly. In differential scanning calorimetry (DSC), **1_C6_** exhibited an LC mesophase over a wide temperature range (<174 °C), while **1_C12_** displayed a phase sequence involving an LC mesophase (35–117 °C) and two crystal phases (–11 to 35 °C and <–11 °C), upon cooling from the corresponding isotropic liquid phases (Fig. 4). Polarized optical microscopy (POM) images of the LC mesophases of **1_C6_** and **1_C12_** showed a fan-shaped texture, which is typically observed for hexagonal columnar (Col_h_) LC assemblies (Fig. 5a and b).

The powder X-ray diffraction (XRD) pattern of a bulk sample of **1_C6_** at 100 °C upon heating displayed five diffraction peaks with *d*-spacings of 1.75, 1.01, 0.88, 0.66 and 0.58 nm. The ratio of the *d*-spacings (1 : √3 : 2 : √7 : 3) completely agrees with the values expected for diffractions from the (100), (110), (200), (210) and (300) planes of a 2D hexagonal lattice (Fig. 5c and S2, ESI†). The hexagonal lattice parameter (*a*), given by 2*d*_100_/√3, was determined to be 2.02 nm. The diffraction peak at scattering vector *q* = 14.6 nm^–1^ (*d*-spacing = 0.43 nm) corresponds to the core-to-core separation of the mesogen along the columnar axis, which is comparable to the bowl-stacking distance in the 1D columns of crystalline **1_C1_** (4.0 Å, Fig. 2). A very broad diffraction peak at approximately *q* = 16 nm^–1^ (*d*-spacing = 0.39 nm) likely arises from the shortest C–C distance in the 1D column, judging from the crystal structure of **1_C1_** (Fig. 2). The diffraction peak corresponding to the core-to-core separation is exceptionally strong and sharp (Fig. 5c and S2, ESI†), compared to those observed for usual discotic columnar LC assemblies.^8^ This observation suggests that the structural order of the columnar arrays of the bowl-shaped mesogen is remarkably high. Although no phase-transition feature was observed below 30 °C in the DSC analysis, temperature-dependent XRD measurements revealed that **1_C6_** undergoes a phase transition from the LC mesophase to a glassy solid phase at approximately 30 °C. Accordingly, the XRD pattern of **1_C6_** at *e.g.*, 20 °C upon cooling (Fig. S2, ESI†) displayed three sharp peaks at a wide-angle region (*q* > 16 nm^–1^). In addition to these peaks, some periodic peaks arising from the ordered Col_h_ structure remained, indicating that the structural feature of the LC mesophase is maintained to some extent in the glassy solid phase.

The structure of **1_C12_** in the LC mesophase was essentially identical to that of **1_C6_**, except for a larger hexagonal lattice parameter (*a* = 2.60 nm) due to the longer alkyl side chains. As shown in Fig. 5d and S3 (ESI†), the XRD pattern of a bulk sample of **1_C12_** at 100 °C upon heating displayed five diffraction peaks with *d*-spacings of 2.26, 1.30, 1.13, 0.75, and 0.42 nm. The last peak, which is assignable to the core-to-core separation of the mesogen, was not affected by the difference in the side-chain lengths. We also note that when branched alkyl chains are attached to the sumanene core, formation of the LC mesophase would be suppressed, by analogy to discotic LC assemblies with a planar aromatic mesogen. For instance, compound **1_EH_** (Fig. 1) only exhibited a phase transition from crystal to isotropic liquid, as revealed by DSC, POM and XRD analyses (Fig. S4, ESI†).

Since molecules in the LC state can behave dynamically, we supposed that the LC sumanenes such as **1_C12_** and **1_C6_** might exhibit a collective bowl-to-bowl inversion in response to electric fields. To explore this possibility, we measured the dielectric properties of bulk samples of **1_C12_** and **1_C6_** (Fig. S5, ESI†). Since dielectric properties are sensitive to the motions of polar molecular units,^9^ dielectric relaxation measurements should detect a thermally activated bowl-to-bowl inversion of **1_C12_** and **1_C6_**, if it occurs. However, the dielectric constants of **1_C12_** and **1_C6_** did not change significantly over a temperature range of crystalline and Col_h_ phases as well as a frequency range of 10^3^–10^6^ Hz (Fig. S5, ESI†), indicating that the LC materials have antiferroelectric properties. Based on this observation, the bowl-stacked 1D columns of **1_C12_** and **1_C6_**, in the Col_h_ mesophases do not respond to the electric fields, but rather statically align in an antiparallel manner to cancel out the dipole generated in each column.

### Complexation of hexathiomethyl sumanene (**1_C1_**) with C_60_

Although sumanene derivatives capable of complexation with C_60_ have never been reported to date, we supposed that this would not be the case with hexathioalkyl sumanenes, since the electron-donating thioalkyl groups could change the inherent electronic properties of sumanene. Indeed, cyclic voltammetry of **1_C1_** in CH_2_Cl_2_ in the presence of tetrabutylammonium hexafluorophosphate as a supporting electrolyte displayed a reversible oxidation wave at *E*_1/2_ = 0.64 V (*versus* ferrocene/ferrocenium), while non-substituted **1_H_** under identical conditions showed an irreversible oxidation wave at 1.03 V (Fig. S6, ESI†). Due to its improved electron-donating properties as well as its concave structure, **1_C1_** might show strong affinity toward electron-accepting C_60_.^10^

The ^1^H NMR spectrum of an equimolar mixture of **1_C1_** and C_60_ ([**1_C1_**] = 1.0 mM, toluene-*d*_8_, 25 °C) showed two doublet signals and one singlet signal at *δ* = 4.51, 3.54, and 2.26 ppm arising from the benzylic *exo*- and *endo*-protons and S–CH_3_ groups of **1_C1_**, respectively (Fig. S7, ESI†). These ^1^H NMR signals were shifted slightly more downfield relative to those observed for **1_C1_** in the absence of C_60_ (4.50, 3.53, and 2.24 ppm, Fig. S7, ESI†). After the NMR measurement, we noticed that a considerable amount of black precipitate formed, which contained both **1_C1_** and C_60_ as confirmed by APCI-TOF mass spectrometric analysis. Obviously, **1_C1_** gave a complex with C_60_. Although the association constant between **1_C1_** and C_60_ could not be determined because of the low solubility of a mixture **1_C1_** and C_60_ in solution, Job's plot^11^ of **1_C1_** with C_60_ in toluene-*d*_8_ at 25 °C (total concentration [**1_C1_**] + [C_60_] = 0.1 mmol), based on the ^1^H NMR chemical shift of the S–CH_3_ signal as a reference, suggested the occurrence of an approximately 1 : 1 complexation (Fig. S8, ESI†), which is most likely due to a concave–convex interaction.^5^

The use of **1_C12_** in place of **1_C1_** allowed the evaluation of the association constant (*K*_a_) with C_60_. When C_60_ was added to a toluene-*d*_8_ solution of **1_C12_** (1.0 mM, [C_60_]/[**1_C12_**] = 0.0–3.7), ^1^H NMR signals due to the benzylic and thioalkyl protons of **1_C12_** gradually shifted downfield without any precipitation (Fig. S9, ESI†). From the ^1^H NMR spectral change, *K*_a_ was determined to be 280 M^–1^ (Fig. S10, ESI†), which is not very high compared to those reported for curved π-systems and C_60_.^2^,4b,f,10b Job's plot of **1_C12_** with C_60_ in toluene-*d*_8_ at 25 °C (total concentration [**1_C12_**] + [C_60_] = 0.1 mmol) confirmed the occurrence of a 1 : 1 complexation (Fig. S11, ESI†).

**Fig. 6:**
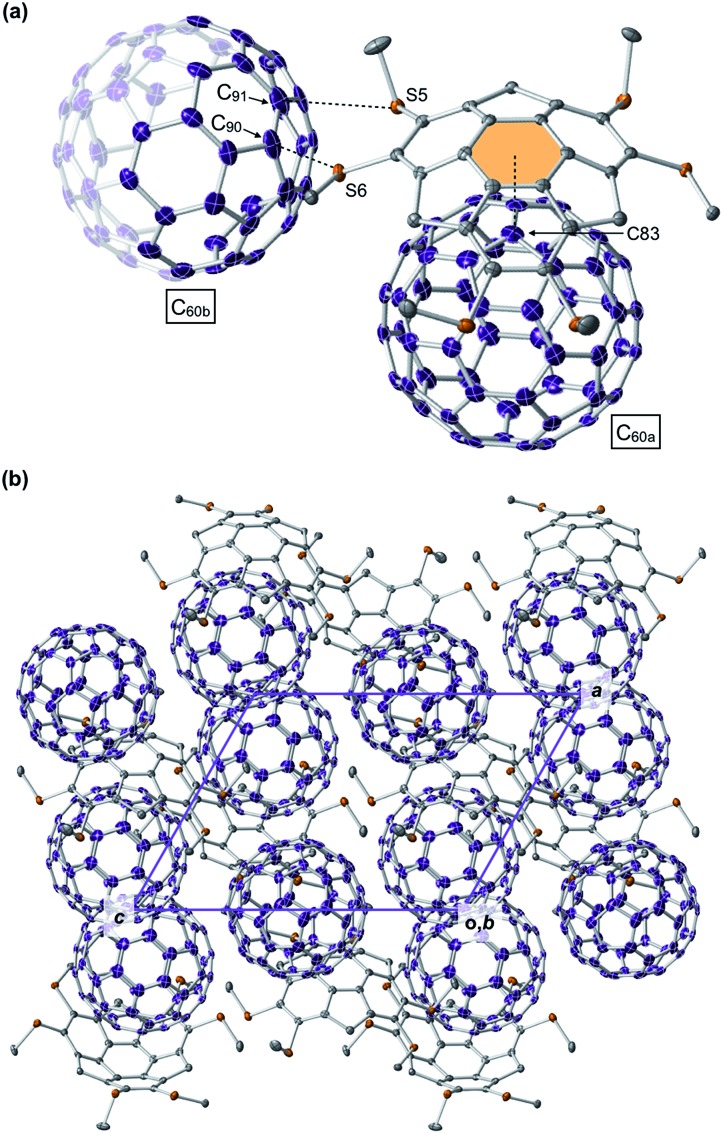
(a) Asymmetric unit and (b) packing diagram in the crystal structure of **1_C1_**·(C_60_)_1.5_. Hydrogen atoms are omitted for clarity. The asymmetric unit involves only half of a C_60b_ molecule, and the remainder is represented translucently. Colour code: carbon (**1_C1_**) = grey, carbon (C_60_) = purple and sulfur = orange.

From a toluene solution of an equimolar mixture of **1_C1_** and C_60_, we successfully obtained a black-colored block single crystal suitable for single-crystal X-ray analysis (ESI†). The X-ray structure showed that **1_C1_** and C_60_ co-crystallized with a ratio of 2 : 3 to constitute a unit cell (space group: triclinic *P*1), where the asymmetric unit contains one **1_C1_** and one and a half C_60_ molecules (Fig. 6). Thus, the complexation stoichiometry of **1_C1_** and C_60_ in the solid state (1 : 1.5) is different from that in solution (∼1 : 1). In the crystal, the two C_60_ molecules are tightly packed without disordering. One of the C_60_ molecules (C_60a_) and **1_C1_** form a concave–convex complex, in which the shortest distance between one of the carbon atoms of C_60a_ (C_83_, Fig. 6a) and the mean plane of the central six-membered ring of **1_C1_** is 3.370 Å. The other C_60_ molecule (C_60b_) interacts with the thiomethyl groups of **1_C1_** (Fig. 6a), as evidenced by the intermolecular sulfur–carbon contacts, *e.g.*, S5–C_90_ (3.164 Å) and S6–C_91_ (3.456 Å), which are shorter than the sum of the van der Waals radii (3.50 Å).^12^ Accordingly, every C_60b_ molecule interacts with a total of 12 thiomethyl groups of six neighboring **1_C1_** molecules, leading to the formation of a two-dimensional network (Fig. S12, ESI†). This structural feature may account for the observed low solubility (*i.e.*, facile crystallization) of the complex of **1_C1_** with C_60_ in solution, despite relatively small association constants between hexathioalkyl sumanenes and C_60_.

## Conclusions

A recently established method for full functionalization of the aromatic rings of sumanene1*i* allowed us to investigate the possibility that these bowl-shaped molecules could act as a mesogen for LC assemblies. As we have demonstrated in the present work, sumanene, when attached to simple thioalkyl side chains, forms a remarkably high-order columnar LC assembly, most likely due to concave–convex interactions. This result is in contrast to those with corannulene with an analogous concave–convex geometry, which requires specific side chains for the formation of a mesophase.2f,g The first successful synthesis of LC sumanenes would be a first step in the development of stimuli-responsive molecular assemblies by taking advantage of the bowl-to-bowl inversion dynamics. We have also demonstrated that, unlike non-substituted sumanene, the hexathioalkyl version behaves as an electron donor, leading to the complexation with C_60_. Since **1_C1_** shows affinity for C_60_, which leads to facile co-crystallization from a dilute solution, hexathioalkyl sumanenes may serve as a new building block for the construction of supramolecular materials with fullerene derivatives.

## Conflicts of interest

There are no conflicts to declare.

## Supplementary Material

Supplementary information

Crystal structure data
