# Supplementary material for: Hexathioalkyl sumanenes: an electron-donating buckybowl as a building block for supramolecular materials
Source: Chem Sci. 2017 Oct 18;8(12):8405–10. doi: 10.1039/c7sc03860g (PMC5863616; doi:10.1039/c7sc03860g)
Supplement: Supplementary file 1 [file SC-008-C7SC03860G-s001.pdf]

Electronic Supplementary Material (ESI) for Chemical Science.
This journal is © The Royal Society of Chemistry 2017

Supplementary Information

Hexathioalkyl sumanenes: An electron-donating buckybowl as a building block for supramolecular materials

Yoshiaki Shoji, Takashi Kajitani, Fumitaka Ishiwari, Qiang Ding, Hiroyasu Sato, Hayato Anetai, Tomoyuki Akutagawa, Hidehiro Sakurai and Takanori Fukushima*

E-mail: fukushima@res.titech.ac.jp (T.F.)

Table of Contents

1. Materials S2
2. General S2
3. Synthesis S2
4. Synchrotron Radiation X-ray Diffraction Experiments S4
5. Single-crystal X-ray Diffraction Analysis S4
6. Theoretical Calculations S5
7. Dielectric Measurements S5
8. Supplementary Tables (Tables S1 and S2) S6
9. Supplementary Figures (Figures S1–S12) S9
10. Supplementary References S20

1. Materials. Unless otherwise stated, all commercial reagents were used as received. Anhydrous tetrahydrofuran (THF) were dried by passage through an activated alumina column and a Q-5 column (Nikko Hansen Co., Ltd.). Non-substituted sumanene[S1] ($\mathbf{1_H}$) and hexabromosumanene[S2] ($\mathbf{1_{Br}}$) were prepared according to the previously reported procedures.

2. General. Recycling preparative size-exclusion chromatography (SEC) was performed using JAIGEL 2H and 2.5H columns on a JAI model LC-9201 recycling HPLC system equipped with a JASCO model MD-2010 Plus variable-wavelength UV-Vis detector with $CHCl_3$ as an eluent. Recycling preparative high-performance liquid chromatography (HPLC) was performed using a KANTO Mightysil Si60 column on a JAI model LC-9201 recycling HPLC system equipped with a JASCO model MD-2010 Plus variable-wavelength UV-Vis detector. NMR spectroscopy measurements were carried out at 25 °C on a Bruker model AVANCE-500 spectrometer (500 MHz for ^{1}H and 125 MHz for ^{13}C) or a Bruker model AVANCE-400 spectrometer (400 MHz for ^{1}H and 100 MHz for ^{13}C). Chemical shifts (δ) are expressed relative to the resonances of the residual non-deuterated solvent for ^{1}H [$CDCl_3$: ^{1}H(δ) = 7.26 ppm, toluene-d_8: ^{1}H(δ) = 7.09, 7.01, 6.97 and 2.08 ppm,] and ^{13}C [$CDCl_3$: ^{13}C(δ) = 78.0 ppm]. Absolute values of the coupling constants are given in Hertz (Hz), regardless of their sign. Multiplicities are abbreviated as singlet (s), doublet (d), doublet of doublets (dd), triplet (t), quartet (q), multiplet (m), and broad (br). Infrared (IR) spectra were recorded at 25 °C on a JASCO FT/IR-660*Plus* Fourier-transform infrared spectrometer. APCI-TOF mass spectrometry measurements were performed on a Bruker model microTOF II mass spectrometer equipped with an atmospheric pressure chemical ionization (APCI) probe. Differential scanning calorimetry (DSC) measurements were carried out using a Mettler–Toledo DSC 1 differential scanning calorimeter, where temperature and enthalpy were calibrated with In (430 K, 3.3 J/mol) and Zn (692.7 K, 12 J/mol) standard samples in sealed Al pans. Cooling and heating profiles were recorded and analyzed using a Mettler–Toledo STAR^e software system.

3. Synthesis.

2,3,5,6,8,9-hexakis(methylthio)sumanene ($\mathbf{1_{C1}}$). Under argon at 25 °C, $\mathbf{1_{Br}}$ (50 mg, 68 μmol) was added to a dry 1,3-dimethyl-2-imidazolidinone (DMI) suspension (3.0 mL) of sodium thiomethoxide (86 mg, 1.2 mmol). The resulting mixture was stirred at 100 °C for 3 h and then allowed to cool to 25 °C. After the addition of CH_3I (0.05 mL), the reaction mixture was stirred at 25 °C for an additional 1 h. The reaction mixture was poured into brine and extracted with CH_2Cl_2. The organic layer separated was washed with water, dried over anhydrous Na_2SO_4, and evaporated to dryness under reduced pressure. The residue, after being washed with MeOH, was subjected to preparative HPLC (Kanto Mightysil column, hexane/CH_2Cl_2 = 2/1 in vol/vol) to allow the isolation of $\mathbf{1_{C1}}$ (13 mg, 24 μmol) as a pale yellow solid in 35% yield: FT-IR (ATR): ν (cm^{-1}) 2976, 2912, 2820, 1628, 1536, 1430, 1387, 1337, 1302, 1231, 1188, 1124, 1011, 961, 904, 826, 727, 691, 634. ^{1}H

NMR (400 MHz, $CDCl_3$, 25 °C): δ (ppm) 4.78 (d, J = 19.0 Hz, 3H), 3.59 (d, J = 19.0 Hz, 3H), 2.61 (s, 18H). ^{13}C NMR (125 MHz, $CDCl_3$, 25 °C): δ (ppm) 150.6, 146.6, 135.0, 43.7, 19.5. APCI-TOF mass: calcd. for $C_{27}H_{24}S_6$ $[M]^+$: m/z = 540.02; found: 540.01.

2,3,5,6,8,9-hexakis(hexylthio)sumanene (1_{C6}). Under argon at 0 °C, hexanethiol (0.22 ml, 1.58 mmol) was added dropwise to a dry THF suspension (2.0 mL) of NaH (38 mg, 1.58 mmol), and the mixture was stirred at 0 °C for 1 h and then evaporated to dryness. Dry DMI (3.0 mL) was added to the residue. Compound $\mathbf{1_{Br}}$ (58.3 mg, 0.079 mmol) was added to the resulting suspension, and the mixture was stirred at 100 °C for 3 h. After being allowed to cool to 25 °C, the reaction mixture was poured into brine and extracted with CH_2Cl_2. The organic layer was washed with water, dried over anhydrous Na_2SO_4, and evaporated to dryness under reduced pressure. The residue was subjected to PTLC (silica gel, *n*-hexane/CH_2Cl_2 = 5/2 in vol/vol) and then GPC ($CHCl_3$) to allow isolation of $\mathbf{1_{C6}}$ (29.6 mg, 0.031 mmol) as a pale yellow solid in 39% yield: FT-IR (ATR): ν (cm^{-1}) 2956, 2925, 2855, 1621, 1536, 1461, 1377, 1335, 1258, 1207, 1231, 1183, 1120, 1048, 1008, 904, 805, 726, 640, 588. ^1H NMR (400 MHz, $CDCl_3$, 25 °C): δ (ppm), 4.68 (d, J = 19.6 Hz, 3H), 7.41 (d, J = 19.6 Hz, 3H), 3.02 (t, J = 7.4 Hz, 12H), 1.73 (m, 12H), 1.47 (m, 12H), 1.31 (m, 24H), 0.89 (t, J = 6.8 Hz, 18H). ^{13}C NMR (125 MHz, $CDCl_3$, 25 °C): δ (ppm) 151.6, 146.6, 134.3, 44.1, 36.3, 31.5, 29.7, 28.6, 22.6, 14.1. APCI-TOF mass: calcd. for $C_{57}H_{84}S_6$ $[M]^+$: m/z = 961.49; found: 961.50.

2,3,5,6,8,9-hexakis(dodecylthio)sumanene (1_{C12}). Using a procedure similar to that for $\mathbf{1_{C6}}$, $\mathbf{1_{C12}}$ was obtained as a pale yellow solid in 39% yield from $\mathbf{1_{Br}}$ and dodecanethiol: FT-IR (ATR): ν (cm^{-1}) 2954, 2924, 2849, 1731, 1625, 1535, 1467, 1428, 1331, 1285, 1256, 1180, 1127, 1006, 907, 802, 719, 643. ^1H NMR (400 MHz, $CDCl_3$, 25 °C): δ (ppm) 4.68 (d, J = 19.9 Hz, 3H), 3.63 (d, J = 19.9 Hz, 3H), 3.02 (t, J = 7.4 Hz, 12H), 1.72 (m, 12H), 1.38–1.21 (m, 96H), 0.88 (t, J = 7.0 Hz, 18H). ^{13}C NMR (125 MHz, $CDCl_3$, 25 °C): δ (ppm) 151.6, 146.6, 134.3, 44.1, 36.3, 31.9, 29.8, 29.7, 29.7, 29.4, 29.4, 29.0, 22.7, 14.1. APCI-TOF mass: calcd. for $C_{93}H_{156}S_6$ $[M + H]^+$: m/z = 1466.06; found: 1466.06.

2,3,5,6,8,9-hexakis(2'-ethylhexylthio)sumanene (1_{EH}). Using a procedure similar to that for $\mathbf{1_{C6}}$, $\mathbf{1_{EH}}$ was obtained as a pale yellow solid in 43% yield from $\mathbf{1_{Br}}$ and 2-ehtylhexylthiol: FT-IR (ATR): ν (cm^{-1}) 2962, 2924, 2864, 1618, 1535, 1459, 1376, 1331, 1285, 1217, 1180, 1112, 1006, 900, 824, 764, 719, 635. ^1H NMR (400 MHz, $CDCl_3$, 25 °C): δ (ppm) 4.70 (d, J =20.2 Hz, 3H), 3.63 (d, J =20.2 Hz, 3H), 3.02 (t, J = 4.6 Hz, 12H), 1.72–1.38 (m, 38H), 1.38–1.21 (m, 24H), 0.88 (m, 36H). ^{13}C NMR (125 MHz, $CDCl_3$, 25 °C): δ (ppm) 151.6, 146.6, 135.0, 44.2, 40.8, 39.7, 32.5, 32.2, 29.0, 28.8, 25.7, 25.4, 23.0, 14.1, 11.1, 10.8. APCI-TOF mass: calcd. for $C_{69}H_{108}S_6$ $[M]^+$: m/z = 1128.68; found: 1128.66.

4. Synchrotron Radiation X-ray Diffraction Experiments. Variable-temperature powder X-ray diffraction (XRD) patterns of the bulk samples of $\mathbf{1}_{\mathbf{C6}}$, $\mathbf{1}_{\mathbf{C12}}$, and $\mathbf{1}_{\mathbf{EH}}$ were obtained using the BL45XU beamline at SPring-8 (Hyogo, Japan) equipped with an R-AXIS IV++ (Rigaku) imaging plate area detector or with a Pilatus3X 2M (Dectris) detector. The scattering vector, $q = 4\pi\sin\theta/\lambda$, and the position of incident X-ray beam on the detector were calibrated using several orders of layer reflections from silver behenate (d = 58.380 Å), where 2θ and λ refer to the scattering angle and wavelength of the X-ray beam (1.0 Å), respectively. The sample-to-detector distances for powder XRD and GI-XRD measurements were 0.50 and 0.44 m, respectively. The obtained diffraction images were integrated along the Debye-Scherrer ring to afford one-dimensional (1D) intensity data using the FIT2D software.[S3] The lattice parameters were refined using the CellCalc ver. 2.10 software.[S4]

5. Single-crystal X-ray Diffraction Analysis. Single crystals of $\mathbf{1}_{\mathbf{C1}}$ (pale yellow needles) and $\mathbf{1}_{\mathbf{C1}}$•$(C_{60})_{1.5}$ (black blocks) were obtained from CH_2Cl_2/hexane and toluene, respectively.

For $\mathbf{1}_{\mathbf{C1}}$, a single crystal was coated with oil base cryoprotectant (Parabar 10312, Hampton Research Corp.) and mounted on a MicroLoops™. Diffraction data were collected at 93 K under a cold nitrogen gas stream on a Rigaku XtaLAB Pro MM007HF X-ray diffractometer system, using multi-layer mirror monochromated Cu-Kα radiation (λ = 1.54187 Å). Intensity data were collected by an ω-scan with 0.5° oscillations for each frame. Bragg spots were integrated, corrected, and scaled using the CrysAlis^{Pro} program package.[S5] Structures were solved by direct methods (SHELXT Version 2014/4)[S6] and refined by full-matrix least squares (SHELXL Version 2016/6).[S7] Anisotropic temperature factors were applied to all non-hydrogen atoms. Hydrogen atoms were placed at calculated positions and refined applying riding models.

For $\mathbf{1}_{\mathbf{C1}}$•$(C_{60})_{1.5}$, a single crystal was coated with immersion oil (type B: Code 1248, Cargille Laboratories, Inc.) and mounted on a micromount. Diffraction data were collected at 90 K under a cold nitrogen gas stream on a Bruker APEX2 platform-CCD X-ray diffractometer system, using graphite-monochromated Mo-Kα radiation (λ = 0.71073 Å). Intensity data were collected by an ω-scan with 0.5° oscillations for each frame. Bragg spots were integrated using the ApexII program package,[S8] and the empirical absorption correction (multi-scan) was applied using the SADABS program.[S9] Structures were solved by a direct method (SHELXT Version 2014/4)[S6] and refined by full-matrix least squares (SHELXL Version 2014/7).[S7] Hydrogen atoms were placed at calculated positions and refined applying riding models.

Crystal data for $C_{27}H_{24}S_6$ ($\mathbf{1}_{\mathbf{C1}}$): pale yellow needles, 0.12 x 0.05 x 0.05 mm^3,trigonal, $P3$, a = 39.0759(5) Å, b = 39.0759(5) Å, c = 7.99482(13) Å, α = 90.000°, β = 90.000°, γ = 120.000°, V = 10572.0(3) Å^3, Z = 18, ρ_{calcd} = 1.529 g cm^{-3}, T = 93 K, $2\theta_{max}$ = 135.4 °, CuKα radiation, λ = 1.54187 Å, μ = 5.491 mm^{-1}, 58781 reflections measured, 25042 unique reflections, 1812 parameters, R_{int} = 0.0444, GOF = 1.085, $R1$ = 0.0831 ($I > 2\sigma(I)$), w$R2$ = 0.2465 (all data),

$\Delta\rho_{min,\ max}$ = –0.46, 1.00 e Å^{-3}, CCDC-1572348.

Crystal data for $C_{117}H_{24}S_6$ (1_{C1}•$(C_{60})_{1.5}$): black blocks, 0.30 x 0.20 x 0.14 mm^3, triclinic, $P\bar{1}$, a = 13.7708(11) Å, b = 16.9234(14) Å, c = 17.1467(14) Å, α = 98.334(1)°, β = 113.332(1)°, γ = 112.891(1)°, V = 3162.1(4) Å^3, Z = 2, ρ_{calcd} = 1.703 g cm^{-3}, T = 90 K, $2\theta_{max}$ = 50.1 °, Mo$K\alpha$ radiation, λ = 0.71073 Å, μ = 0.288 mm^{-1}, 23130 reflections measured, 11101 unique reflections, 1125 parameters, R_{int} = 0.0315, GOF = 1.014, $R1$ = 0.0381 ($I > 2\sigma(I)$), w$R2$ = 0.0958 (all data), $\Delta\rho_{min,\ max}$ = –0.36, 1.01 e Å^{-3}, CCDC-1572349.

6. Theoretical Calculations. Density functional theory (DFT) calculations were performed using Gaussian 09 program package.[S10] Geometry optimizations of $\mathbf{1}_{\mathbf{C1}}$ and $\mathbf{1}_{\mathbf{H}}$ (singlet states in vacuum) were performed using the w97BD functional with a basis set of 6-311G++(d,p). Cartesian coordinates and energies of the computed structures are listed in Tables S1 and S2.

7. Dielectric Measurements. The temperature-dependent dielectric constants of $\mathbf{1}_{\mathbf{C6}}$ and $\mathbf{1}_{\mathbf{C12}}$ were measured using a two-probe AC impedance method at frequencies from 1 kHz to 1 MHz (Hewlett-Packard, HP4194A), where temperature was controlled by a Linkam model LTS-E350 temperature control system. Sumanenes $\mathbf{1}_{\mathbf{C6}}$ and $\mathbf{1}_{\mathbf{C12}}$ were placed in a liquid crystal cell (KSSZ-05/A111P6NSS05 from E. H. C Co., Ltd.) having indium tin oxide electrodes (1 cm^2 in area) with a gap of 5 μm. The LC samples were annealed at 473 K under vacuum while applying a DC voltage of 5 V.

8. Supporting Tables.

Table S1. Optimized Cartesian coordinates (in Angstrom) of $\mathbf{1}_{C1}$ (a singlet state in vacuum) at the w97BD/6-311G++(d,p) level. Total energy is −3432.38102464 hartree.

C	0.409700	1.345000	1.152400
C	-0.934200	1.047200	1.155900
C	-1.858700	1.851500	0.499800
C	-1.404900	3.062400	-0.017100
C	0.000000	3.389400	0.010800
C	0.925700	2.477800	0.528600
C	2.414700	2.195100	0.233900
H	2.664500	2.436900	-0.801900
H	3.094200	2.769400	0.871900
C	-1.369600	-0.317700	1.152400
C	-0.439800	-1.332600	1.155900
C	-0.674100	-2.535400	0.499800
C	-1.949700	-2.747900	-0.017100
C	-2.935300	-1.694700	0.010800
C	-2.608700	-0.437200	0.528600
C	-3.108300	0.993600	0.233900
H	-3.442700	1.089100	-0.801900
H	-3.945500	1.295000	0.871900
C	0.959900	-1.027300	1.152400
C	1.373900	0.285400	1.155900
C	2.532700	0.683900	0.499800
C	3.354600	-0.314500	-0.017100
C	2.935300	-1.694700	0.010800
C	1.682900	-2.040600	0.528600
C	0.693700	-3.188700	0.233900
H	0.778200	-3.526000	-0.801900
H	0.851200	-4.064400	0.871900
S	4.821100	0.163100	-0.919400
S	3.975100	-2.886200	-0.801600
S	-2.269300	-4.256700	-0.919400

S	-4.487000	-1.999500	-0.801600
S	-2.551800	4.093600	-0.919400
S	0.511900	4.885600	-0.801600
C	3.651100	-4.388900	0.166200
H	4.494600	-5.052400	-0.028300
H	3.623100	-4.154300	1.230900
H	2.732600	-4.891400	-0.133700
C	6.072800	-0.134700	0.366100
H	5.856400	0.457600	1.255300
H	6.109400	-1.196200	0.613200
H	7.032500	0.173200	-0.051200
C	1.975400	5.356400	0.166200
H	2.869700	4.812200	-0.133700
H	2.128200	6.418600	-0.028300
H	1.786200	5.214800	1.230900
C	-2.919800	5.326600	0.366100
H	-2.018800	5.889000	0.613200
H	-3.666200	6.003700	-0.051200
H	-3.324500	4.843000	1.255300
C	-5.626400	-0.967500	0.166200
H	-5.602300	0.079200	-0.133700
H	-6.622800	-1.366300	-0.028300
H	-5.409200	-1.060500	1.230900
C	-3.153100	-5.191900	0.366100
H	-3.366200	-6.176900	-0.051200
H	-2.531900	-5.300600	1.255300
H	-4.090600	-4.692800	0.613200

Table S2. Optimized Cartesian coordinates (in Angstrom) of $\mathbf{1}_{\mathbf{H}}$ (a singlet state in vacuum) at the w97BD/6-311G++(d,p) level. Total energy is −807.33745221 hartree.

C	-1.243000	0.656200	-0.701800
C	-0.085200	1.403000	-0.701700
C	0.000000	2.639900	-0.069000

C	-1.185700	3.150800	0.453600
C	-2.381900	2.378900	0.453600
C	-2.405100	1.088200	-0.069000
C	-3.277600	-0.161800	0.185100
H	-1.194300	4.094400	0.990100
H	-3.238100	2.775900	0.989900
H	-3.663700	-0.180500	1.206700
H	-4.137500	-0.203800	-0.492200
C	1.189800	0.748400	-0.701800
C	1.257600	-0.627800	-0.701700
C	2.286200	-1.319900	-0.069000
C	3.321500	-0.548600	0.453600
C	3.251200	0.873300	0.453600
C	2.144900	1.538800	-0.069000
C	1.498700	2.919400	0.185100
H	4.143000	-1.012900	0.990100
H	4.023000	1.416400	0.989900
H	1.675500	3.263100	1.206700
H	1.892300	3.685000	-0.492200
C	0.053300	-1.404600	-0.701800
C	-1.172500	-0.775300	-0.701700
C	-2.286200	-1.319900	-0.069000
C	-2.135800	-2.602200	0.453600
C	-0.869300	-3.252300	0.453600
C	0.260200	-2.627000	-0.069000
C	1.778900	-2.757600	0.185100
H	-2.948700	-3.081500	0.990100
H	-0.784900	-4.192200	0.989900
H	1.988200	-3.082600	1.206700
H	2.245200	-3.481300	-0.492200

9. Supplementary Figures.

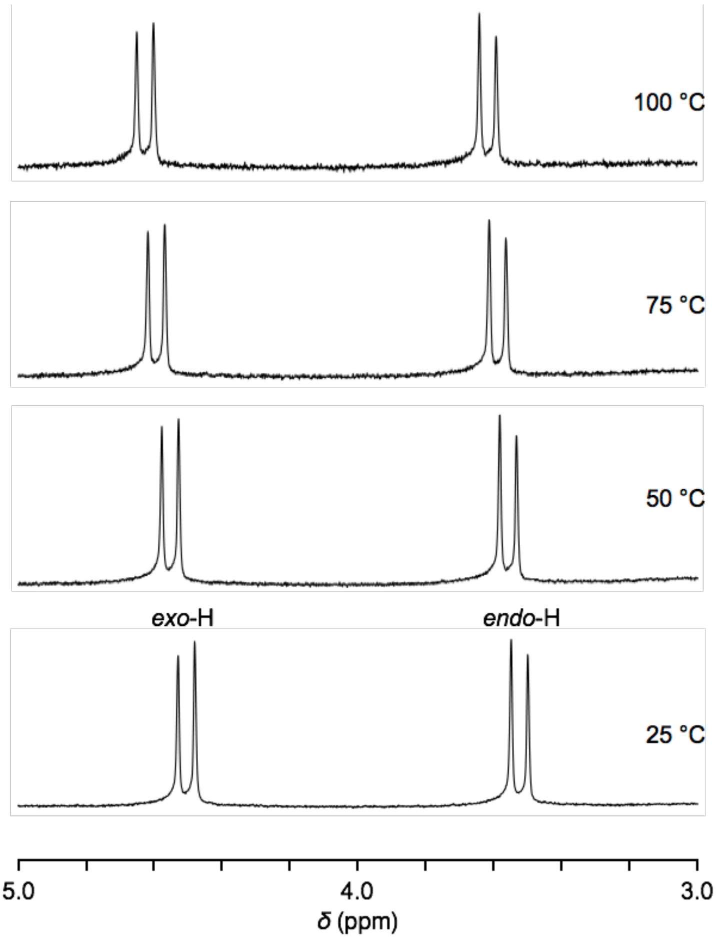

Figure S1. Variable-temperature ^{1}H NMR spectra (400 MHz) of $\mathbf{1}_{\mathbf{C1}}$ in toluene-d_8.

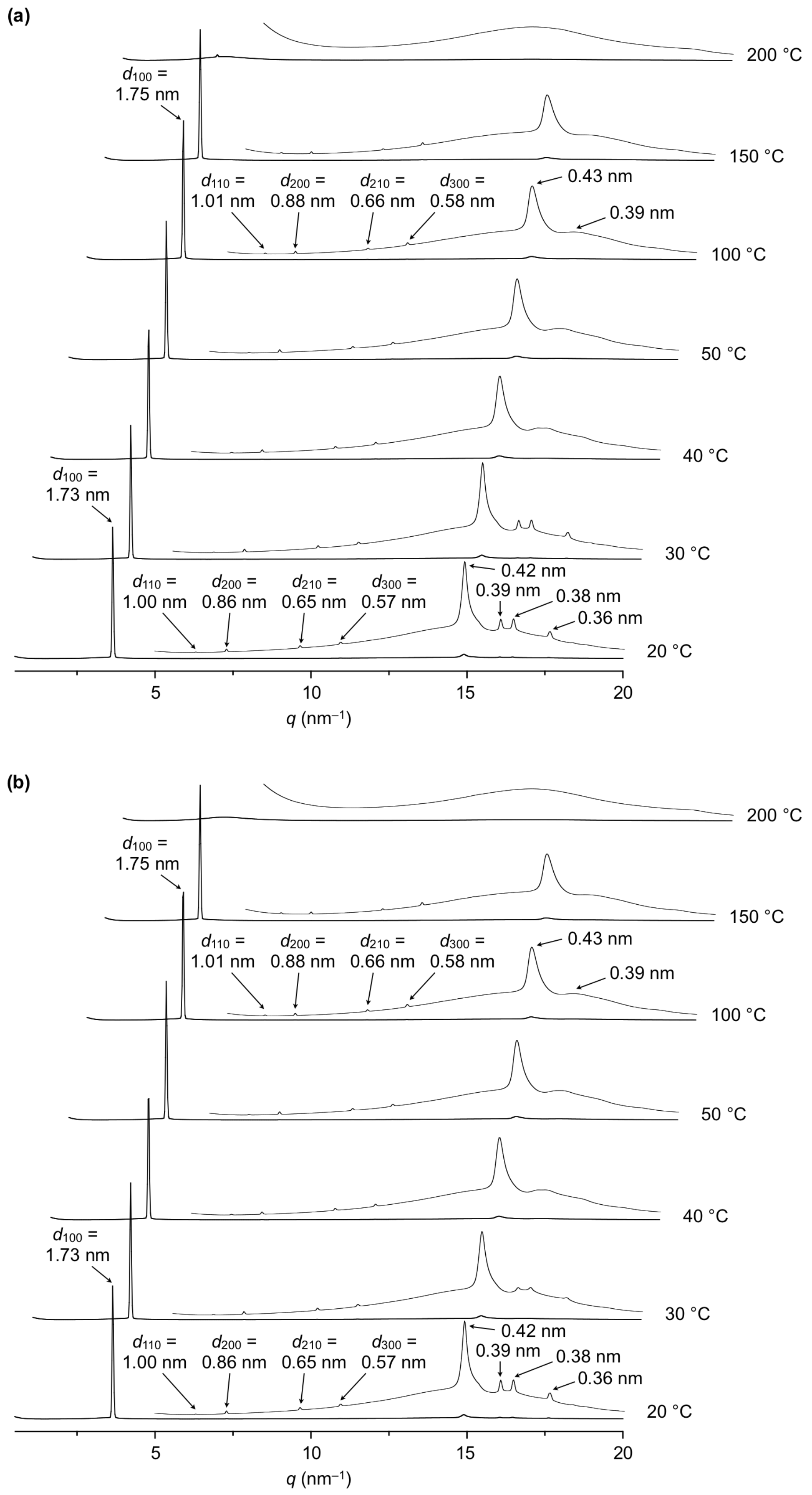

Figure S2. Powder XRD patterns and their peak assignments of a bulk sample of $\mathbf{1}_{\mathbf{C6}}$ at 25 °C on (a) cooling and (b) heating in a glass capillary (1.5 mm-ϕ).

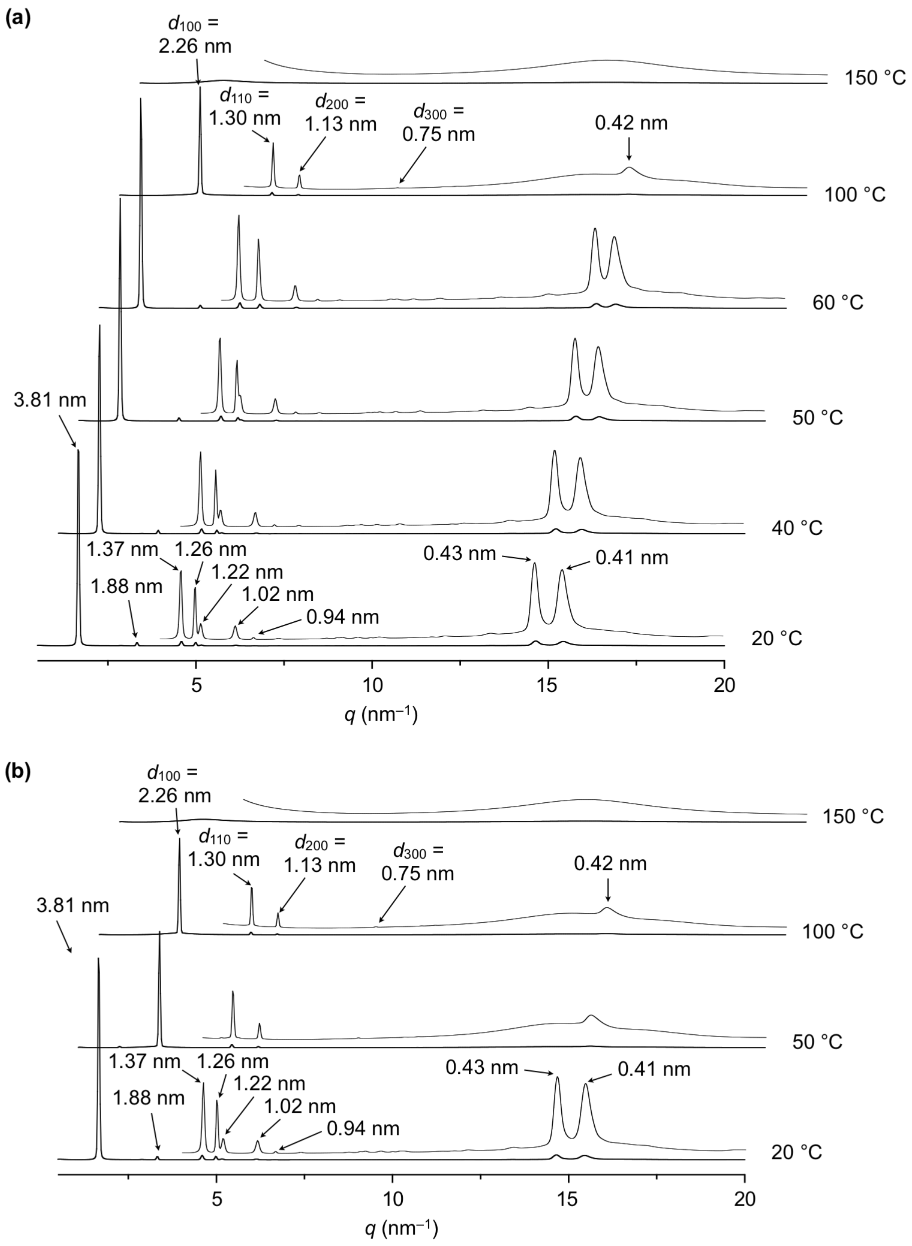

Figure S3. Powder XRD patterns and their peak assignments of a bulk sample of $\mathbf{1}_{\mathbf{C12}}$ at 25 °C on (a) cooling and (b) heating in a glass capillary (1.5 mm-ϕ).

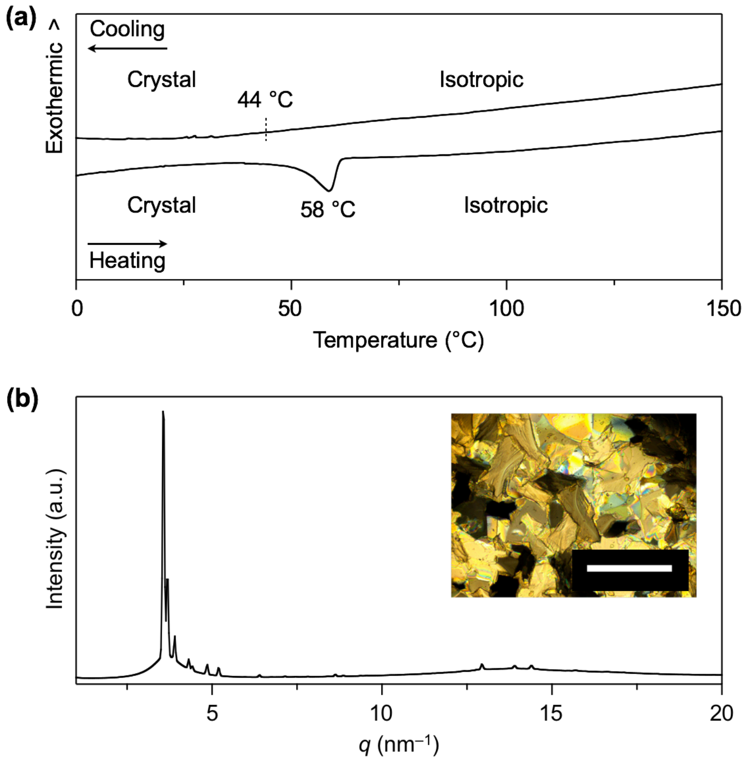

Figure S4. (a) DSC profile at the second heating/cooling cycle of $\mathbf{1}_{\mathbf{EH}}$. (b) Powder XRD pattern of a bulk sample of $\mathbf{1}_{\mathbf{EH}}$ at 30 °C on cooling from its isotopic melt in a glass capillary (1.5 mm-ϕ). Inset: POM image of $\mathbf{1}_{\mathbf{EH}}$ at 30 °C on cooling from its isotopic melt (scale bar = 500 μm).

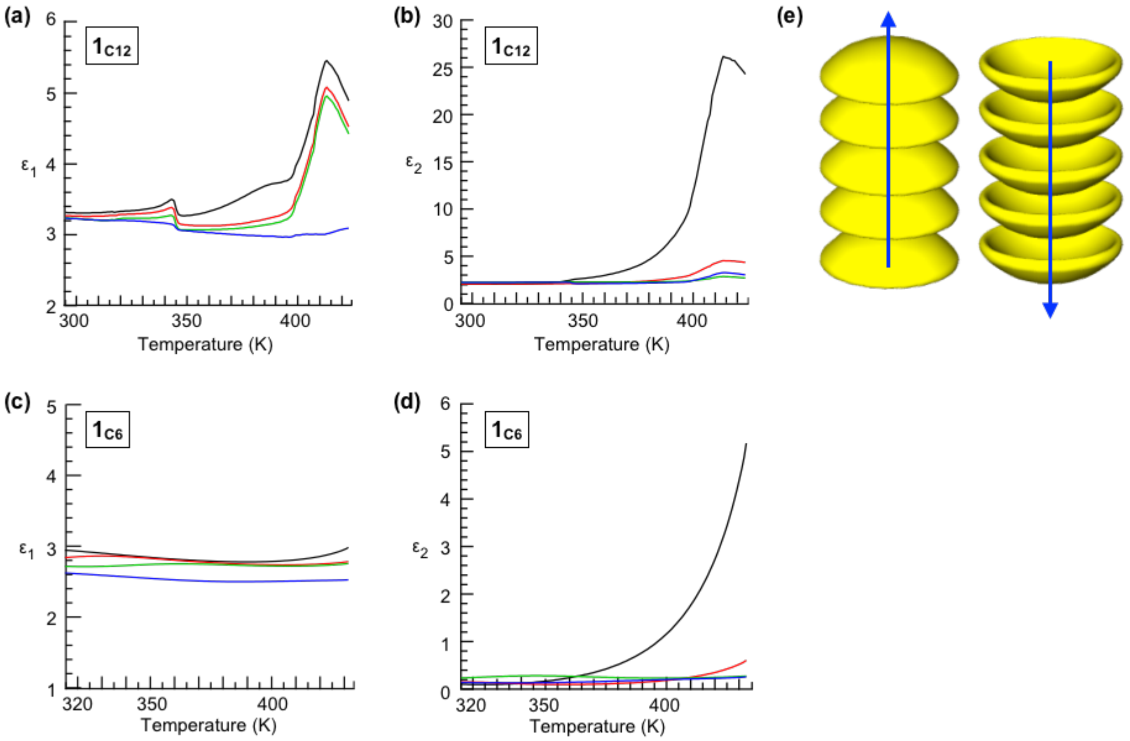

Figure S5. (a) Temperature- and frequency-dependency of the real part of dielectric constants (ε_1) for **1**$_{\mathbf{C12}}$. At every frequency, a subtle dielectric anomaly is observed around the crystal-to-Col$_h$ phase transition temperature (at 343 K based on DSC), and the ε_1 values of the crystal phase (T < 340 K) are almost constant (~3) without frequency dependence. The ε_1 value gradually increased as the temperature increased above 360 K at the frequencies below 100 KHz. The dielectric peak observed around 395 K is associated with the phase transition from the Col$_h$ phase to isotropic liquid. The absence of a dielectric peak at the frequency of 1 MHz means that the molecular motion of **1**$_{\mathbf{C12}}$ is slower than 100 kHz in the LC mesophase. Regardless of frequencies (< 100 kHz), a significant increase in the dielectric constant was observed upon heating up to ~430 K, indicating an antiferroelectric arrangement in the Col$_h$ phase as schematically illustrated in (e). (b) Temperature- and frequency-dependency of an imaginary part of dielectric constants (ε_2) of **1**$_{\mathbf{C12}}$. As in the case of ε_1, the ε_2 value gradually increased as the temperature increased above 360 K at the frequencies below 100 KHz. The dielectric peak observed around 395 K is associated with the phase transition from the Col$_h$ phase to isotropic liquid. At 10 kHz, a relatively large ε_2 value (~24) observed at 410 K is likely due to the occurrence of electrical conduction in the Col$_h$ phase. (c and d) Temperature- and frequency-dependency of real (ε_1) and imaginary (ε_2) parts of dielectric constants for **1**$_{\mathbf{C6}}$, respectively. Although the dielectric responses are similar to those observed for **1**$_{\mathbf{C12}}$, the dielectric peak associated with the Col$_h$-to-isotropic liquid phase transition (at 451 K based on DSC) was not observed, presumably due to a thermal decomposition under the measurement conditions. (e) Schematic illustration of antiferroelectrically arranged bowl-stacked 1D columns of the sumanene core.

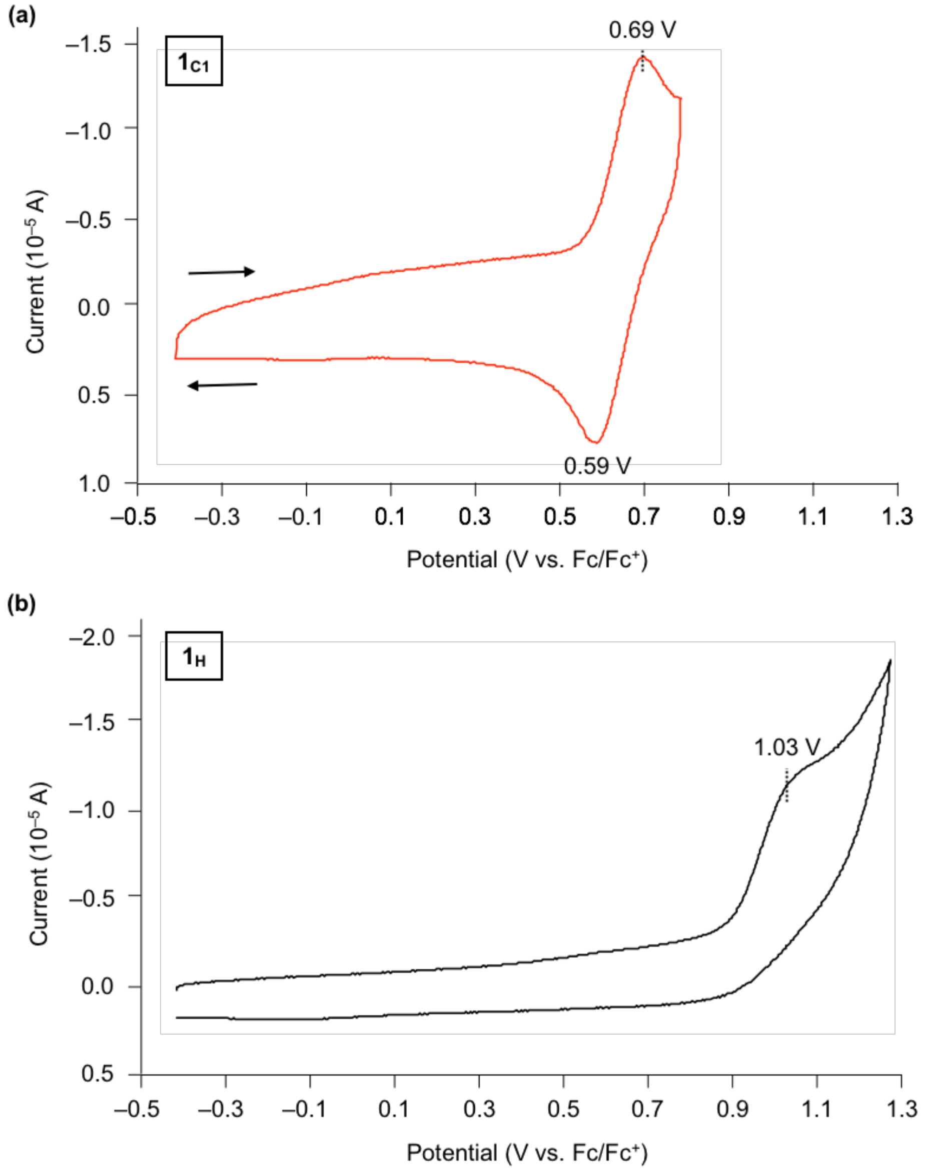

Figure S6. Cyclic voltammograms of (a) $\mathbf{1}_{\mathrm{C1}}$ (1.0×10^{-4} M) and (b) $\mathbf{1}_{\mathrm{H}}$ (1.0×10^{-4} M) in CH_2Cl_2, containing $[Bu_4N^+][PF_6^-]$ (0.1 M) as the supporting electrolyte. Working electrode: glassy carbon; counter electrode: Pt wire; pseudo-reference electrode: Ag wire; scan rate: 100 mV/s.

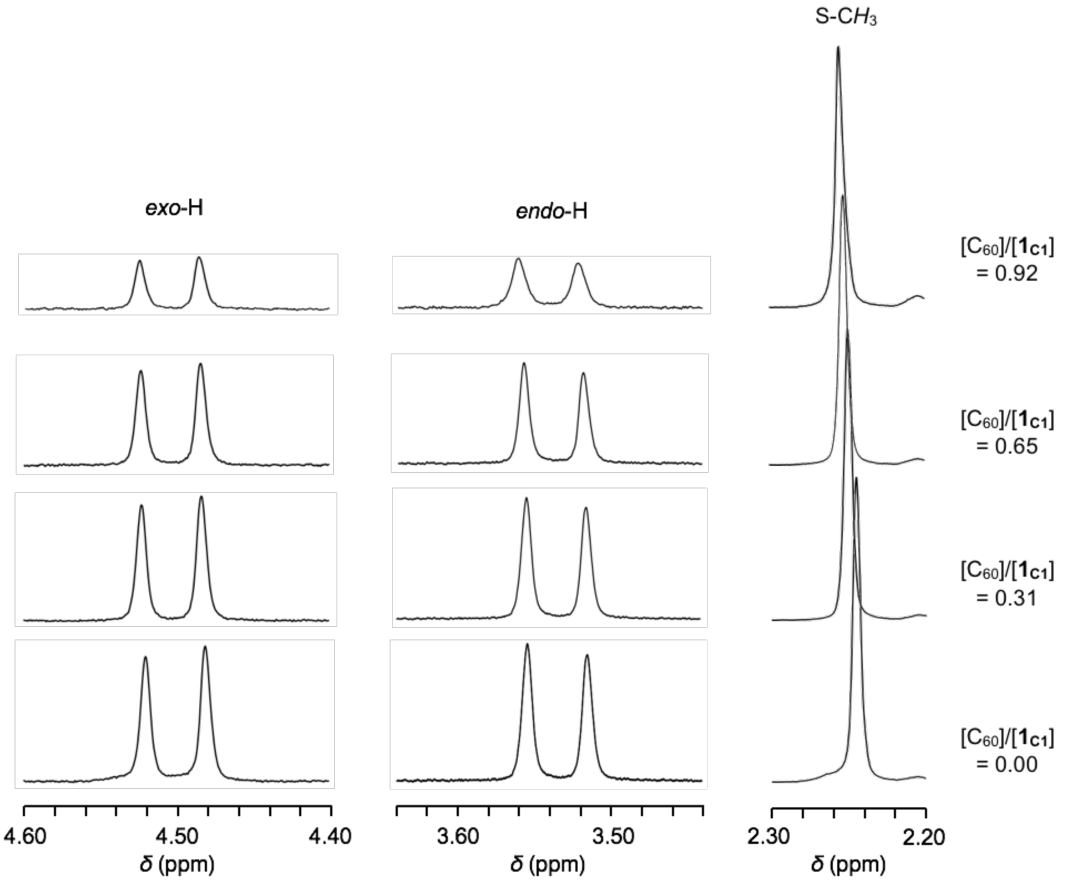

Figure S7. ^{1}H NMR spectra of mixtures of $\mathbf{1}_{\mathbf{C1}}$ and C_{60} in toluene-d_8 at 25 °C. [$\mathbf{1}_{\mathbf{C1}}$] = 1.0 mM. [C_{60}]/[$\mathbf{1}_{\mathbf{C1}}$] = 0.00–0.92.

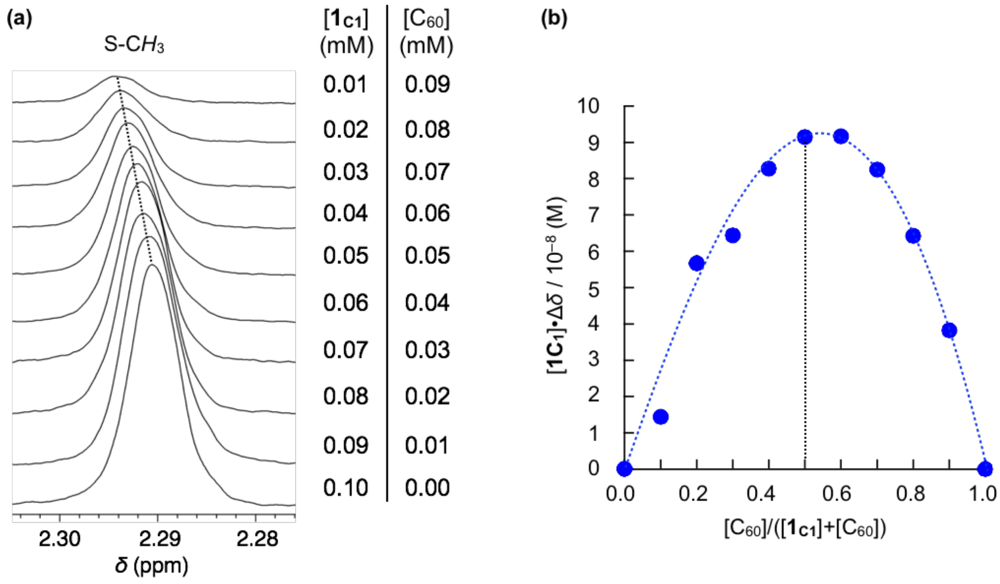

Figure S8. (a) ^{1}H NMR spectra (S-CH_3 region) of mixtures of $\mathbf{1}_{\mathbf{C1}}$ and C_{60} ([$\mathbf{1}_{\mathbf{C1}}$]+[C_{60}] = 0.1 mM) in toluene-d_8 at 25 °C. [C_{60}]/([$\mathbf{1}_{\mathbf{C1}}$]+[C_{60}]) = 0.0 to 0.9. (b) Job's plots using the $\Delta\delta$ values of the S-CH_3 signal.

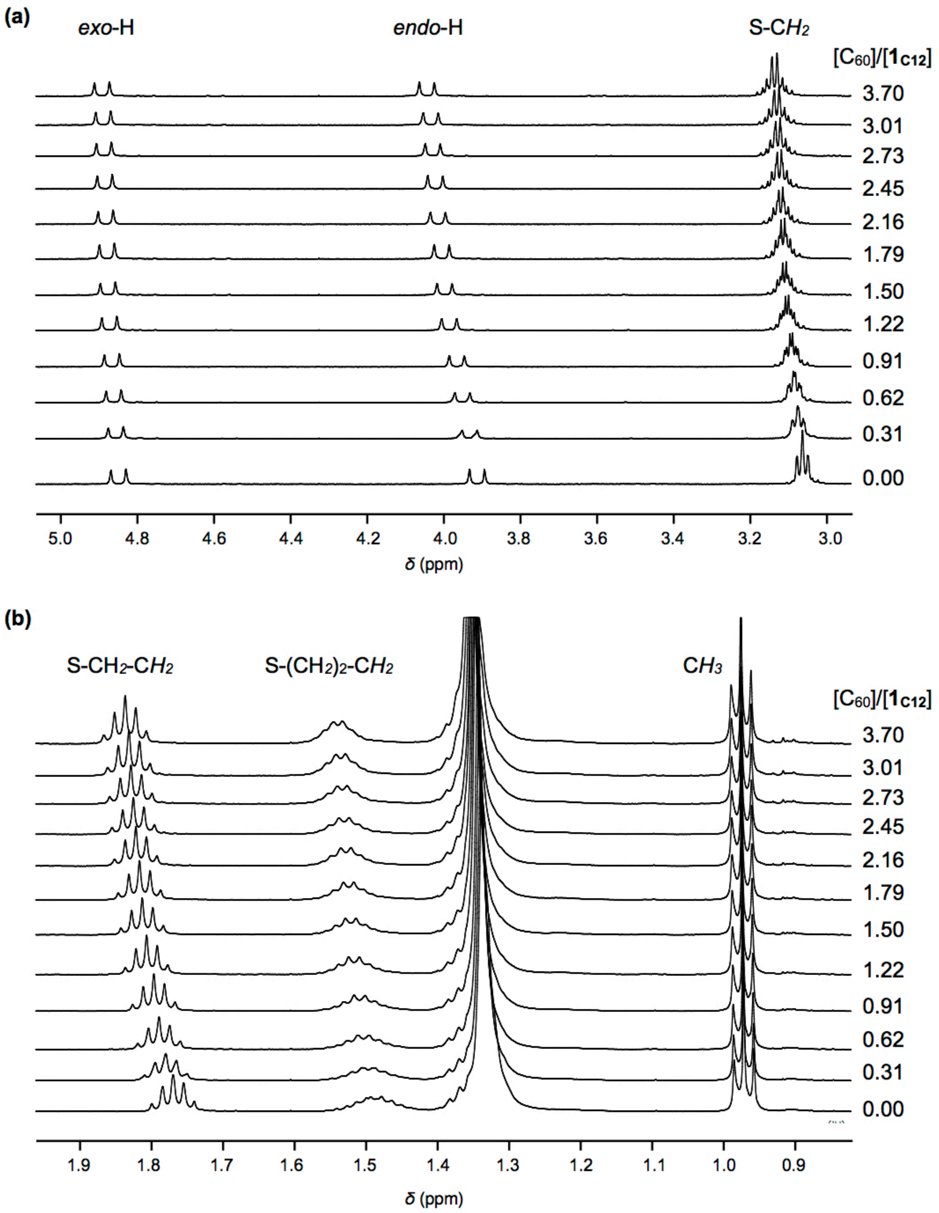

Figure S9. ^{1}H NMR spectral change of $\mathbf{1}_{C12}$ ([$\mathbf{1}_{C12}$] = 1.0 mM) in ranges of (a) 5.0–3.0 ppm and (b) 1.9–0.9 ppm upon titration with C_{60} ([C_{60}]/[$\mathbf{1}_{C12}$] = 0.00–3.70) in toluene-d_8 at 25 °C.

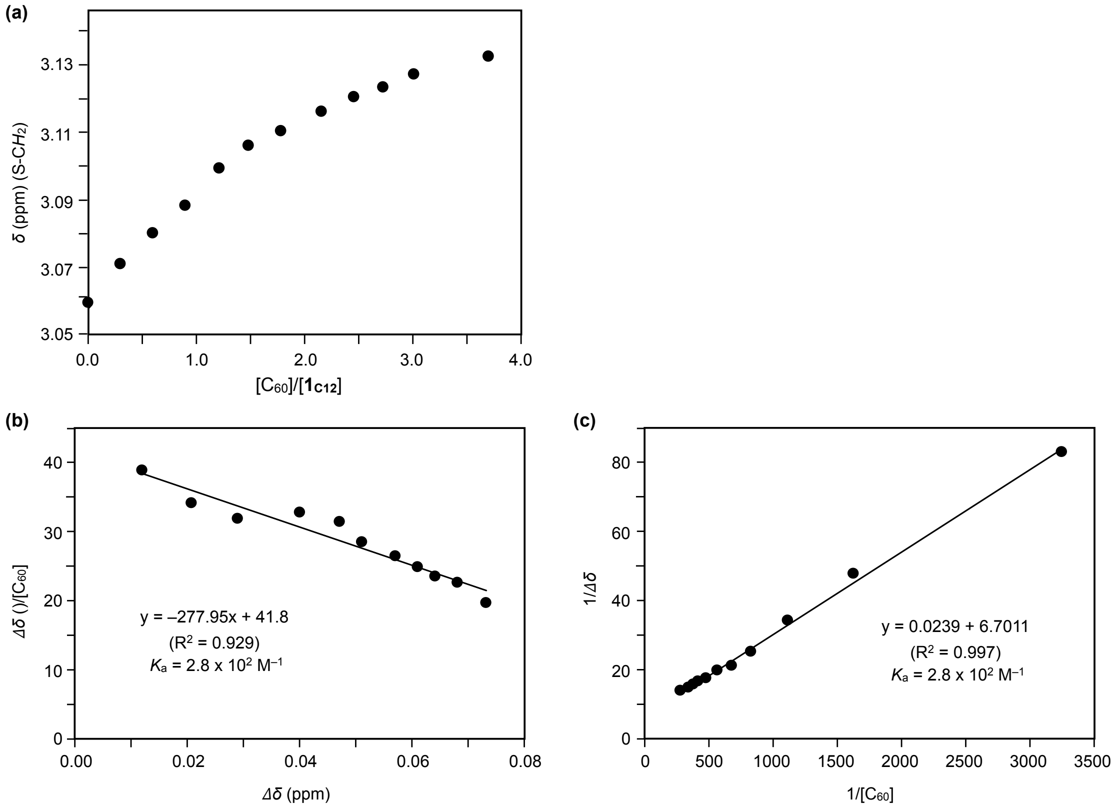

Figure S10. (a) Plots of the ^{1}H NMR chemical shift (δ) of the S-CH_2 groups of $\mathbf{1}_{C12}$ versus [C_{60}]/[$\mathbf{1}_{C12}$] (0.00–3.70). The data were obtained from Fig. S9a. (b) Foster-Fyfe plot[S11] and (c) Benesi-Hildebrand plot[S12] using the $\Delta\delta$ values in (a).

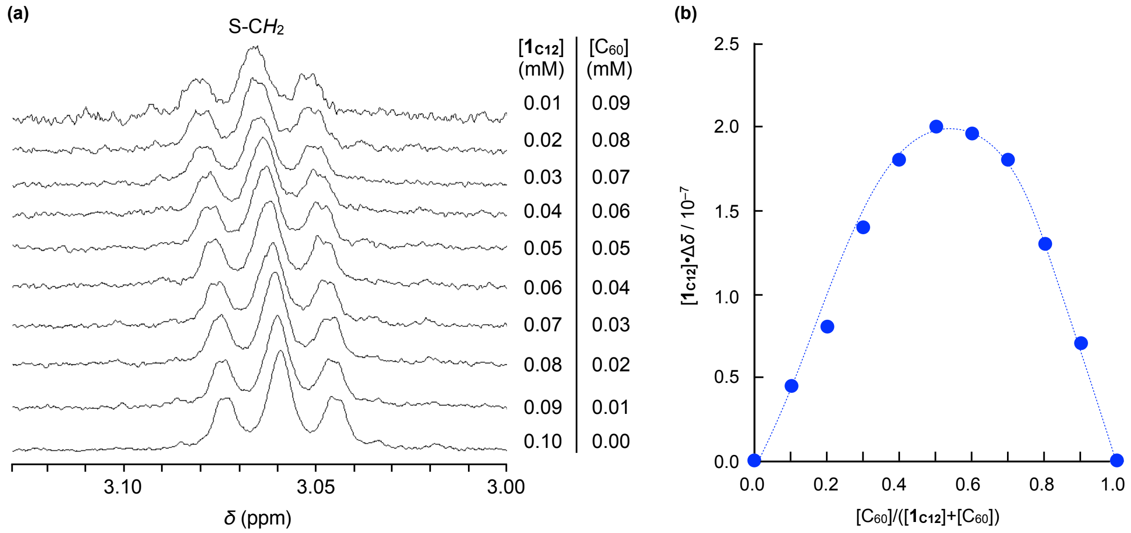

Figure S11. (a) ^{1}H NMR spectra (the S-CH_2 region) of mixtures of $\mathbf{1}_{C12}$ and C_{60} ([$\mathbf{1}_{C12}$]+[C_{60}] = 0.1 mM) in toluene-d_8 at 25 °C. [C_{60}]/([$\mathbf{1}_{C12}$]+[C_{60}]) = 0.0 to 0.9. (b) Job's plot using the $\Delta\delta$ values of the S-CH_2 signal.

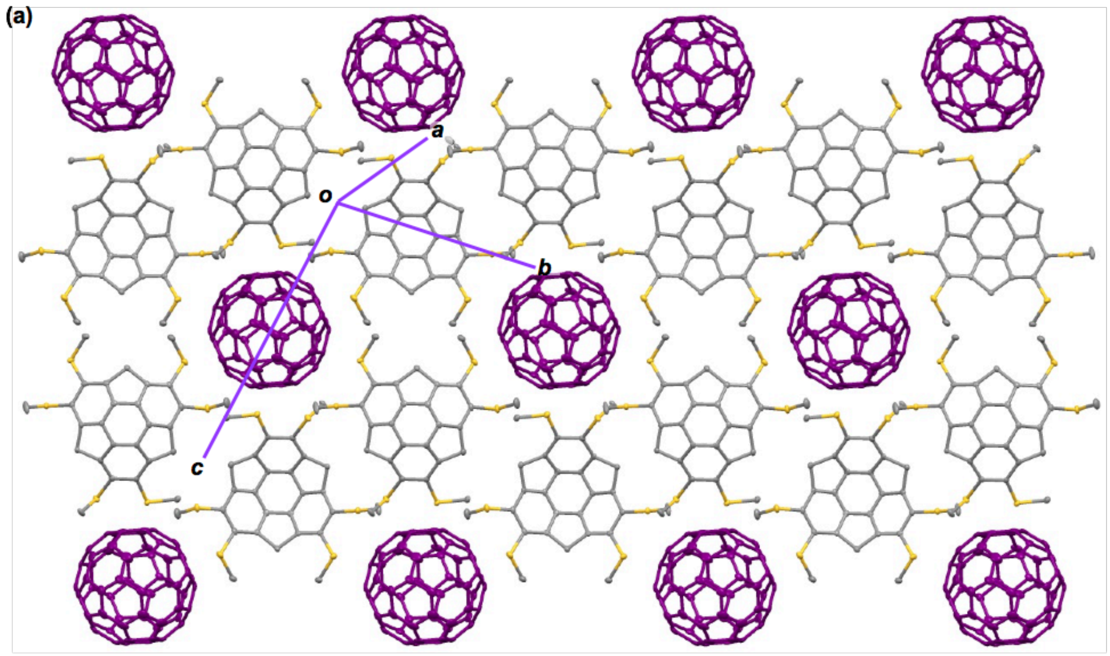

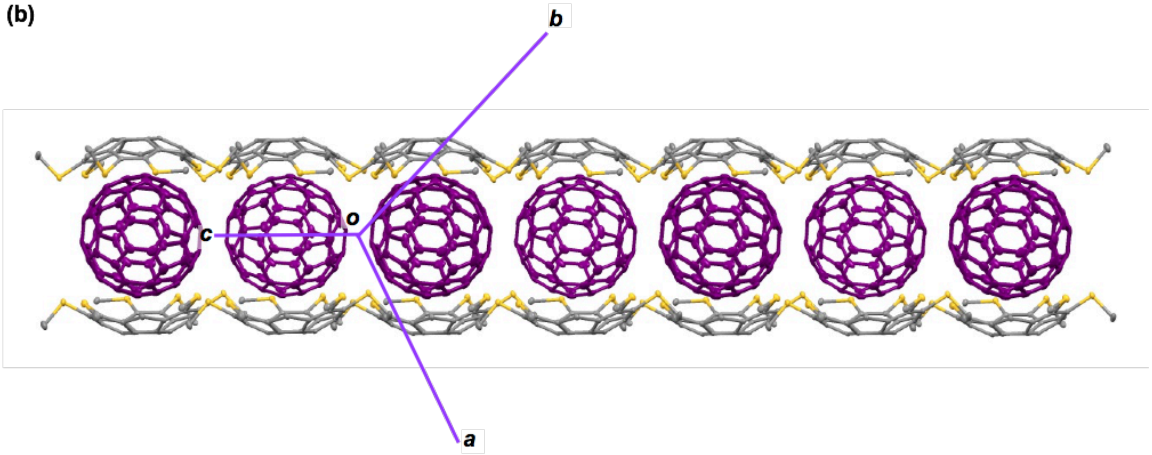

Figure S12. Two-dimensional network formed by $\mathbf{1}_{\mathbf{C1}}$ and the C_{60b} molecules in the crystal (see also Fig. 6). (a) Top view and (b) side view. The C_{60a} molecules and hydrogen atoms are omitted for clarity.

10. Supplementary References

S1. H. Sakurai, T. Daiko, T. Hirao, *Science*, 2003, **301**, 1878.

S2. H. Toda, Y. Yakiyama, Y. Shoji, F. Ishiwari, T. Fukushima, H. Sakurai, *Chem. Lett.*, 2017, **46**, 1368.

S3. FIT2D: http://www.esrf.eu/computing/scientific/FIT2D/

S4. CellCalc ver. 2.10: http://homepage2.nifty.com/~hsc/soft/cellcalc_e.html

S5. Rigaku Oxford Diffraction, CrysAlisPro, Rigaku Corporation, Tokyo, Japan. 2015.

S6. G. M. Sheldrich, *Acta Cryst*. 2015, ***A71***, 3.

S7. G. M. Sheldrich, *Acta Cryst*. 2015, ***C71***, 3.

S8. *APEX 2,* version 2011.11-3, Bruker AXS Inc., Madison, Wisconsin, USA.

S9. L. Krause, R. Herbst-Irmer, G. M. Sheldrick, D. Stalke, *J. Appl. Crystallogr.* 2015, **48**, 3.

S10. M. J. Frisch, G. W. Trucks, H. B. Schlegel, G. E. Scuseria, M. A. Robb, J. R. Cheeseman, G. Scalmani, V. Barone, B. Mennucci, G. A. Petersson, H. Nakatsuji, M. Caricato, X. Li, H. P. Hratchian, A. F. Izmaylov, J. Bloino, G. Zheng, J. L. Sonnenberg, M. Hada, M. Ehara, K. Toyota, R. Fukuda, J. Hasegawa, M. Ishida, T. Nakajima, Y. Honda, O. Kitao, H. Nakai, T. Vreven, J. A. Montgomery, J. E. Peralta, Jr., F. Ogliaro, M. Bearpark, J. J. Heyd, E. Brothers, K. N. Kudin, V. N. Staroverov, R. Kobayashi, J. Normand, K. Raghavachari, A. Rendell, J. C. Burant, S. S. Iyengar, J. Tomasi, M. Cossi, N. Rega, J. M. Millam, M. Klene, J. E. Knox, J. B. Cross, V. Bakken, C. Adamo, J. Jaramillo, R. Gomperts, R. E. Stratmann, O. Yazyev, A. J. Austin, R. Cammi, C. Pomelli, J. W. Ochterski, R. L. Martin, K. Morokuma, V. G. Zakrzewski, G. A. Voth, P. Salvador, J. J. Dannenberg, S. Dapprich, A. D. Daniels, O. Farkas, J. B. Foresman, J. V. Ortiz, J. Cioslowski, D. V. Fox, Gaussian 09 (Revision A.02), Gaussian, Inc., Wallingford CT, 2009.

S11. R. Foster, C. A. Fyfe, *J. Chem. Soc., Chem. Commun.*, 1965, 642.

S12. H. Tsukube, H. Furuta, A. Odani, Y. Takeda, Y. Kudo, Y. Liu, H. Sakamoto, K. Kimura, In *Comprehensive Supramolecular Chemistry*, J. E. D. Davies, J. A. Ripmeester, Eds., *Elsevier Science*, Oxford, 1996, **8**, pp 425–482.
